# Supplementary material for: Are carbon emissions trading and green financial instruments synergistic? -Comprehensive quantitative research based on content analysis
Source: PLoS One. 2024 Mar 7;19(3):e0298601. doi: 10.1371/journal.pone.0298601 (PMC10919601; doi:10.1371/journal.pone.0298601)
Supplement: S1 Data — (ZIP) [file pone.0298601.s001.zip › Data/地级市Z_F工作报告.zip!/║■─╧/╥μ╤⌠╩╨/└┤╫╘╣1⁄2╓┌║┼í╛┬φ┐╦╩2╛▌═°í┐_2003.docx]

各位代表：

　　现在，我代表市人民政府，向大会作政府工作报告，请予审议，并请各位政协委员和其他列席人员提出意见。

　　一、过去五年工作的回顾

　　益阳市第二届人民代表大会第一次会议以来，在中共益阳市委的正确领导下，在市人大、政协的监督支持下，本届政府高举邓小平理论伟大旗帜，全面贯彻“三个代表”重要思想，紧紧依靠全市人民，解放思想，实事求是，与时俱进，开拓创新，国民经济和社会发展取得了明显成效。

　　（一）综合经济实力不断增强。五年来，国内生产总值年均增长8.2%，全社会固定资产投资年均增长11%，社会消费品零售总额年均增长7.6%，财政总收入年均增长8.3%。经济结构调整成效显著。农业和农村经济结构调整取得成效，乡镇企业、劳务经济、避灾农业、畜牧水产业和农机产业稳步发展，农业产业化步伐加快。传统工业增速提质，规模工业实现整体扭亏为盈，园区经济加快发展，益鑫泰公司、沅江纸业公司、南洲酒业公司、建玲竹业公司、新兴铸管公司、橡塑机公司等一批骨干企业发展壮大，引进了纳爱斯公司、科力远公司、惠同新材料公司、中科软件南方公司、汉森制药公司、中宇爱斯柯公司等一批高新技术企业和民营科技企业。商贸物流、旅游休闲、社区服务等第三产业迅速发展。

　　（二）基础设施建设成效显著。五年来固定资产投资累计达255亿元。洛湛铁路益娄段、益常高速公路、益桃一级公路、东小公路、白沙大桥、安化资江大桥建成通车，茅草街大桥、桃江资江大桥、益沅一级公路正在抓紧建设。通信建设日新月异，信息化水平不断提升。实施洞庭湖二期治理、水库处险保安和平垸行洪、移民建镇工程，投入各类水利建设资金45.2亿元，投入劳动工日4.3亿个，移动土石方4.5亿方，防灾抗灾能力大为增强。益阳电厂一期工程竣工投产，城乡电网改造顺利进行。城镇化步伐加快，城镇化水平由27.5%提高到31%。中心城区扩容与提质并举，主干道骨架初步形成，建成了资江三桥、奥林匹克公园、沿江风光带、梓山湖高尔夫球场和一批城市广场，污水处理厂建设进展顺利，市容市貌明显改观，人居环境进一步改善。中心城区5年完成建设投资8.2亿元，建成区面积达到40平方公里，人口达到40万。全市共投入小城镇基础设施建设资金11.5亿元，小城镇面貌发生了新的变化。

　　（三）改革开放取得新进展。农村双层经营体制进一步巩固和完善，土地流转和适度规模经营有序进行，农村税费改革取得阶段性成果。国有农场撤场建区改革基本完成。国有企业改革进行了积极探索，清塘煤矿、医药公司、肉联厂、制药厂等一批国企改革取得成功。政府机构和行政审批制度改革取得新的进展。劳动力市场逐步形成，就业再就业和社会保障工作进一步落实。财政、金融、住房等各项改革不断深化,整顿规范市场经济秩序的工作成效明显。招商引资力度加大，对外开放和区域经济技术合作领域不断扩大，成功举办了’99中国竹文化节和2003年国际竹文化研讨会，益阳知名度进一步提高。

　　（四）社会事业全面进步。五年来，先后有39个重点项目进入国家级科技计划，95个重点项目进入省级科技计划，获省级科技进步奖励25项，科技进步对经济增长的贡献率有新的提高。“普九”水平不断提高，高中阶段教育加快发展，民办教育有了新的进步。高等教育取得突破，成功组建湖南城市学院，正在筹建益阳职业技术学院和湖南工艺美术职业学院。体育事业发展迅速。建成了规格较高的体育设施；成功举办了湖南省第九届运动会和第六届残疾人运动会，益阳代表团分别获得金牌总数第三和第一的成绩；承办了全国第五届城市运动会羽毛球和女子足球两项比赛。计划生育工作水平提高，继续保持全省先进单位称号。国土矿产资源管理、生态建设、环境保护力度加大，资江整治取得阶段性成果。文化、卫生、民政、民族、宗教、工商、质量技术监督、药品监督、审计、统计、外事、侨务、对台、气象、水文、地震、人防、无线电管理、残联、科协、老年科协、档案、地方志、老龄等各项工作有了新的进展。工会、共青团、妇联在现代化建设中发挥了积极作用。

　　驻益人民解放军、武警部队的现代化建设取得新成绩，国防后备力量和民兵预备役建设不断加强。在全面建设小康社会，特别是抗击非典、防汛抗旱和消防抢险中作出了重要贡献。

　　（五）民主法制建设和精神文明建设得到加强。认真执行人大及其常委会决议、决定，自觉接受人大法律监督。积极支持政协及各民主党派、工商联参政议政和民主监督。共办理人大代表建议、批评、意见605件，政协委员提案1302件，办复率100%。全面推行政务、厂务、村务公开，村民和居民自治等基层民主建设取得新的成绩，社区建设步伐加快。加强普法教育，群众民主法制观念进一步增强。行政执法、行政执法监督、行政复议应诉得到加强。严厉打击严重刑事犯罪和经济犯罪，强化社会治安综合治理，加强信访工作，确保了全市社会政治大局的稳定。精神文明建设成效显著，深入学习“三个代表”重要思想、党的十六大和十六届三中全会精神，高度重视思想政治工作和公民道德建设，广泛开展以创“十星级农户”、“五星级居民户”为主要内容的群众性精神文明创建活动和文化活动，市民素质得到提高。

　　（六）人民生活不断改善。五年来，城镇居民人均可支配收入年均增长7.8%，农民人均纯收入年均增长5.6%。城乡居民储蓄存款余额比1998年增加1.8倍。“两个确保”目标基本实现，下岗职工基本生活保障费和企业离退休人员基本养老金实现了按时足额发放。扶贫工作取得较大成绩。广播、电视的人口覆盖率分别比1998年提高46个和34个百分点，城乡人民文化生活不断丰富，生活质量有所提高。救灾救济工作有序开展，受灾群众和特困群众的基本生活得到保障。

　　2003年，是全市人民开拓进取、奋力拼搏的一年，是迎难而上、加速发展的一年，全市经济发展呈现出增长比较快、效益比较好、后劲比较强的特点，市第二届人民代表大会第五次会议确定的目标基本实现，确定的任务基本完成。一是经济工作重点进一步突出。按照确定的工作思路，以项目立市为突破口，全力加强项目建设，千方百计增加固定资产投资。扎扎实实抓了水利建设，提高防洪抗灾能力。狠抓中心城区建设，改造了秀峰湖公园，启动了会龙山公园的改造，建设了会志路、陆贾山路、秀峰中路、教育路、海棠路、鹿角园路、资阳大道，全面推进县城和重点镇的扩容提质，努力提高城镇化水平。突出抓好园区建设，精心打造经济亮点，启动了益阳高新区南扩20平方公里的建设工程。抓好招商引资，把招商引资作为发展益阳经济的重要途径。努力发展壮大民营经济，把发展民营经济作为发展市域、县域经济的一个战略举措来抓，坚决走经济以民营为主，投资以民间为主，事业以民办为主的发展道路。全力推动劳务经济发展，劳务收入成为农民增收的主要来源之一。二是加快经济发展的措施进一步强化。继续推进经济结构调整。调整区域经济结构，初步形成了以益阳城区为中心，银桃、银沅、银龙三线13个镇为重点的城镇发展体系。以农民增收为目标，调整农村经济结构，重点扶持了农业产业化龙头企业和优质农产品基地建设。以加快工业化进程为重点，调整产业结构。以做大做强名优产品为重点，调整产品结构。全面推进国有企业改革。出台了《关于加快国有企业改革的决定》、《市直国有企业理顺职工劳动关系和离退休人员社会保障关系的实施办法》、《市直国有企业产权处置的实施办法》等文件，成立了国企改革领导小组，并从市直机关部门抽调精兵强将组成30个指导小组，由市级领导带队，进驻第一批改革的30家市直国有企业指导改革。加强财源建设，狠抓财税征管，完成了财政收入全年目标任务。全力做好招商引资工作。出台了《益阳市鼓励投资的若干规定》，深洽会益阳招商周签约项目64个，合同引进资金74.5亿元。创建金融安全区，银行加大对经济支持的力度。银行与企业融资洽谈会，签订贷款合同62个、22.8亿元，到目前50%的资金已到位。出台了《关于对各区县（市）完成今年各项经济工作任务情况进行考核的通知》，进一步强化工作责任，加强组织领导，使各项工作落到实处。着力培育旅游市场，旅游经济健康发展。坚持以人为本，认真解决关系人民群众切身利益的问题。全力抓了防治非典工作，取得了防治非典的阶段性重大胜利。继续推进人口与资源、环境的可持续发展战略，退耕还林、退田还湖、农业综合开发、农村能源建设工作取得明显成效。“两南林业”快速发展。平垸行洪、移民建镇稳步推进，6500户农民移居新址，安居乐业。千方百计扩大就业，努力改善创业和就业环境；抓紧健全各项社会保障制度，继续做好“两个确保”工作，抓好失业保险和城市居民最低生活保障制度的落实，搞好“三条保障线”的衔接；抓好救灾救济工作，认真抓好安全生产。三是政府自身建设进一步加强。狠抓政府职能转变。坚持勤政为民，全面推进依法行政，牢固树立新的政绩观，进一步优化经济发展环境。集中时间和精力，深入基层，深入实际，解决具体问题。大力精简控制会议，提高政府工作效率，严格规范办文程序，坚持少发文，少开会。廉洁自律，从严治政，切实做到勤政、严政、廉政。四是经济发展速度进一步加快。2003年，预计完成国内生产总值240亿元（老口径统计为253.1亿元），比上年增长9.4％。财政总收入11.37亿元，增长5.1％。全社会固定资产投资60.7亿元，增长11％。社会消费品零售总额90.5亿元，增长9.9％。进出口总额9000万美元，增长25.8％，其中出口6000万美元，增长119%；实际利用外资5000万美元，增长9.9%；利用内资19亿元，增长25.3%。城镇居民人均可支配收入7400元，增长8.8%；农民人均纯收入2300元，增长4.8%。金融运行平稳，金融机构存贷款余额分别达到190.3亿元和153.7亿元，比年初分别增长14%和12%。

　　各位代表！过去五年的成绩来之不易，是全市人民同心同德、顽强奋斗的结果。在此，我代表市人民政府，向全市人民，向人大代表、政协委员，向各民主党派、工商联、各人民团体、社会各界人士，向中央和省驻益单位、人民解放军和武警部队官兵，向所有参与、支持和关心益阳发展的海内外朋友，表示崇高的敬意和衷心的感谢！

　　在充分肯定成绩的同时，必须清醒地看到，在全市经济和社会发展过程中仍存在着不少矛盾和困难。主要是：制约经济发展的体制性障碍比较突出，经济结构还不合理，工业化程度低；财政收入规模小，支出压力大，特别是县乡财政困难；下岗失业人员和新增需要就业的人员多，就业矛盾突出；少数群众生活比较困难，农民收入和部分城镇居民收入增长缓慢；教育、卫生防疫、医疗保健等事业投入不足，应对突发性公共卫生事件能力不强；经济发展环境、社会治安、市场秩序等方面还需进一步改善；政府职能转变与新形势、新任务的要求还不相适应。必须高度重视并切实解决这些问题。

　　二、今后五年的奋斗目标和2004年工作重点

　　今后五年是我市加快发展、全面建设小康社会的关键时期，也是向第三步战略目标迈进的重要时期。以党的十六大和十六届三中全会召开为标志，我国改革开放和现代化建设进入了新的发展阶段。我国经济正处于经济周期的上升阶段，进入了新一轮增长期。展望下一个五年的发展，我们充满了信心。经过改革开放特别是撤地建市以来的快速发展，我市综合经济实力明显增强，为加快发展奠定了坚实的基础；我市资源丰富，区位优势明显，发展潜力巨大；全市人心思进，加快益阳发展已成为全市人民的强烈愿望和共同意志。只要我们乘势而上，奋发努力，完全可以发挥后发优势，加快发展步伐。

　　今后五年，我市经济工作的总体思路是：坚持第一要务，突出两个重点（硬环境建设和软环境建设），实施五大战略（工业强市、项目立市、开放活市、科教兴市和依法治市战略），加快“三化”（工业化、农业产业化、城镇化）进程，为益阳全面建设小康社会打下坚实基础。我市经济社会发展的奋斗目标是：力争全市国内生产总值和财政总收入年均递增10%以上，到2008年全市国内生产总值突破400亿元，财政总收入突破20亿元，全社会固定资产投资5年累计达到500亿元，农民人均纯收入年均递增5%以上，城镇居民人均可支配收入年均递增7%以上，人口自然增长率控制在7‰以内。

　　今后五年，在经济工作中要牢牢把握以下几条原则：

　　一是既要紧紧抓住经济建设这个中心，更要走全面协调可持续发展的道路。要进一步突出经济建设这个中心，唱响经济发展的主旋律，一切工作服从服务于这个中心，千方百计加快经济发展；同时要按照统筹城乡发展、区域发展、经济社会发展、人与自然和谐发展以及对内发展与对外开放的要求，树立全面、协调、可持续的发展观，坚持统筹兼顾，加强协调发展，保持经济持续快速协调健康发展和社会全面进步。

　　二是既要加快经济发展速度，更要提高经济增长的质量和效益。坚持速度与效益的统一，既要牢牢把握发展主题，保持经济的较快发展，确保在全省经济格局中占据应有的地位，更要深化改革，调整结构，提高经济增长的质量和效益，使全市经济发展的速度，是有效益的速度，是财政增长的速度，是人民增收的速度。

　　三是既要抓好基础设施建设，更要突出抓好产业建设。要把基础设施建设作为产业发展的重要前提和条件，继续抓好交通、能源、通信、水利建设。要切实把经济工作的重点放在产业建设上，突出发展先进制造业，形成以高新技术产业为先导、基础产业和制造业为支撑、服务业全面发展、具有益阳特色的产业格局。

　　四是既要高度重视农业，抓好农村经济，更要突出抓好工业，走工业强市之路。要以农民增收为核心，着力解决“三农”问题，实现农业和农村经济稳定健康发展。要把加快工业发展，作为增强益阳综合实力、提高发展水平的重点来抓，扶持壮大一批骨干企业，大力发展中小企业，加速推进工业化进程，迅速壮大工业经济规模，实现工业强市的目标。

　　五是既要抓好高新技术产业的发展，更要突出抓好用先进适用技术改造提升传统产业。要在抢占经济“制高点”，掌握发展主动权，大力发展高新技术产业的同时，立足现有基础，发挥比较优势，整合资源，盘活存量，全力改造提升传统产业，加快传统产业的优化升级。

　　做好2004年的工作，对加快我市经济社会发展，保证“十五”计划全面实施至关重要。2004年政府工作的总体要求是：坚持以邓小平理论和“三个代表”重要思想为指导，全面贯彻落实十六大、十六届三中全会和市第三次党代会精神，坚持以人为本，树立全面、协调、可持续的发展观，深化改革，扩大开放，优化发展环境，加快发展速度，提高发展水平，增强发展后劲，着力推进“三化”进程，实现国民经济持续快速协调健康发展和社会全面进步。主要预期目标是：国内生产总值增长9.5%以上，全社会固定资产投资增长20%，社会消费品零售总额增长10%，实际利用外资增长20%，进出口总额增长20%，财政总收入增长10%，城镇居民人均可支配收入增长8%，农民人均纯收入增长6%，城镇登记失业率控制在5%以下，人口自然增长率控制在6‰以内。

　　为了实现预期目标，2004年要重点抓好以下八个方面的工作：

　　（一）大力实施工业强市战略，切实加快工业化进程

　　坚持走新型工业化道路，提升工业的整体素质和水平。力争工业增加值增长16%以上，产销率达到98.5%；规模工业产值突破85亿元，利润突破1.5亿元；全市完成技术改造投资突破10亿元。

　　进一步加大扶优扶强的力度。以资本为纽带，优势企业为龙头，推动产业重组，提高产业集中度，培育一批主业集中、主导产品突出、掌握核心技术和自主知识产权、拥有知名品牌的先进制造业大型企业，重点培育沅江纸业公司、益鑫泰公司、科力远公司、汉森制药公司、纳爱斯公司、橡塑机公司等企业，把建材、造纸、食品加工、机械制造等优势产业做大做强。

　　高度重视发展传统产业。积极引进、鼓励投资传统产业，加快传统优势产业的规模扩张，重点是走好资产重组、资本运营的路子。搞好传统优势产品的适用性、开发性调整，多出优品、精品，加快更新换代，使传统产品以质取胜。积极引导和扶持企业建立技术创新体系，鼓励企业与高等院校、科研院所通过联合研究、委托开发、成果转化，共建研究开发机构和科技型企业实体。整合资源，加强技改，大力改造提升纺织、锑品、竹业、农机等重点传统产业，逐步形成支撑益阳工业发展的支柱产业。同时，鼓励支持中小企业发展。

　　大力发展高新技术产业。抓紧实施益阳高新技术产业开发区南扩20平方公里的规划，积极申报国家火炬计划湖南先进制造业产业基地，加快建设湖南省高等院校科研院所成果转化产业开发基地，积极引进高新技术企业。大力培育和发展科力远高能电池、中科软件存储器、惠同新材料、益鑫泰生物面料、华昌纳米级锑品等电子信息、新材料、绿色纺织品、节能环保以及生物工程为主体的高新技术产品。大力发展园区经济，切实加快益阳新技术产业开发区、龙岭工业园、长春工业园等现有园区建设步伐。

　　（二）认真解决“三农”问题，千方百计增加农民收入

　　毫不动摇地巩固和加强农业基础地位，高度重视并着力解决农业、农村和农民问题，把增加农民收入作为农村工作的中心任务和基本目标，按照多予、少取、放活的方针，采取更直接、更明确、更有力的措施，把农业和农村内部的文章做足。

　　大力调整农业产业结构。加强农业服务体系建设，引导农民调整种养业结构、品种结构和区域布局，大力发展高效经济作物和畜牧水产业，大力发展“两南”林业，培育具有竞争力的农产品品牌，构建优势农产品产业带。建立健全农产品检验检测体系和认证体系，逐步实行市场准入制度。大力发展无公害农产品、绿色食品和有机食品，新建扩建一批无公害和绿色农产品标准化生产示范基地与生态农业示范基地。依托现有产业化龙头企业，建设200万亩优质稻基地、200万亩南抗杨基地、100万亩“双低”油菜基地、30万亩棉花基地、80万亩蔬菜基地、50万亩优质亚麻苎麻基地，扩建名特优水产品生产基地和优质茶生产基地。

　　积极推进农业产业化经营。大力发展农产品加工业，健全龙头企业与农民风险共担、利益共享的机制。重点扶持粒粒晶、益鑫泰、油中王、辣妹子、泰升果汁、沅江纸业、建玲竹业、桃花江竹胶板、谊信肉食、益华水产等一批带动性强、覆盖面广的产业化龙头企业，延长农业产业链，提高农产品附加值和市场占有率。积极引导重点龙头企业完善经营机制，提高科技创新能力，增强核心竞争力。培育壮大农村专业合作经济组织、市场中介组织和行业协会，积极推广专业户带农户、公司+基地+农户、公司+中介组织+农户、订单农业等方式，提高农民进入市场的组织化程度。

　　大力发展劳务经济。继续推进劳动力外出就业，全方位开辟农民就业和增收的渠道。加强组织培训，搞好信息服务，规范劳务中介市场，加强地区之间劳务协作，扩大劳务输出规模，提高劳务输出的质量和效益。保护外出务工经商农民的合法权益。

　　加强农业执法，严厉打击各类坑农害农行为，维护农民利益。加大农业综合开发力度，大力推进以沼气开发为主的农村能源建设和生态环境建设。全面完成平垸行洪、移民建镇工作。

　　（三）努力扩大投资消费需求，不断提高城镇化水平

　　认真实施项目立市战略。科学制定产业规划，继续抢抓机遇，加大投资力度，全力以赴抓项目。加强项目库建设，组建专门班子，集中力量谋划一批符合国家产业政策和投资方向的重点项目，进一步完善2004－2008年全市重大建设项目库。加强对项目准备、项目争取和项目实施工作的组织领导，认真落实重点项目领导联系制度、定期会商制度，强化各级各部门项目争取责任制，定期调度检查，严格奖惩兑现。主动加强同上级有关职能部门的衔接，争取更多的项目进入国家和省里的“笼子”。完成桃江资江大桥建设和县城及大通湖区电网改造工程，建好高新区、马公铺两座110千伏变电站，加快茅草街大桥、沅江赤山大桥、白沙港口、小马公路、县乡公路改造等项目建设进度，争取早日动工建设益阳电厂二期工程、绕城线、资江四大桥，尽快开工建设南县麻河口大桥、常吉高速公路安化联络线、益阳汽车南站等项目，做好沅江黄茅洲大桥、小河口大桥、益阳至芦林潭千吨级航道等项目的前期准备工作。建立村级电子阅览室，全力支持政府上网和企业上网，利用无线技术解决农村通信普遍服务问题，实现全市村村通电话。加强水利建设，突出抓好湖区的大堤加修和山丘区病险水库、山塘的处理，沅江、赫山、资阳全面完成两水夹堤吹填任务，南县完成60%，进一步提高防汛抗灾能力。

　　努力提高第三产业发展水平。大力发展商贸物流业，鼓励发展连锁经营、大型综合商场等新兴商业业态，培育和扶持大型连锁企业的发展，推动连锁经营向城市社区和农村集镇延伸。大力发展旅游业，启动创建“中国优秀旅游城市”工程，继续实施旅游精品战略，着力打造梓山湖核心景区、桃花江风景区、南洞庭湖湿地等拳头产品，创建全国农业旅游示范点和国家4A级风景区。改造提升餐饮、娱乐、宾馆、交通运输等传统服务业，积极发展家政、装修、保洁、保安等社区服务业，加快发展金融、证券、通信、会展等现代服务业，规范发展会计、律师、公证、监理、咨询、评估等中介服务业。引导居民增加住房、教育、电信、保健、汽车等方面的消费，形成新的消费热点，扩大消费需求。

　　突出抓好中心城区的扩容提质。按照“南扩东接北改”的思路，坚持高起点规划，高标准建设，高效能管理，高水平经营，着力提升中心城区的辐射带动功能。抓紧进行城市总规修编，认真搞好分区规划及专业规划，精心编制好龙洲路、朝阳路、梓山湖主题公园和高新区南扩详细规划；加大规划执法力度，强化规划监管。加强城市基础设施和配套设施建设，建设五福路、玄宫路等6条道路，拉通大桃南路和秀峰东路，配套完善罗溪南路、东环路和桃花仑东路延伸线，改造好三里桥广场，综合治理好幸福渠，完成垃圾综合处理场地下设施建设，建成污水处理厂并投产运营，抓好城市防洪工程建设；继续搞好绿化、亮化、美化提质建设，打造更多的精品和亮点。房地产开发实现小区化、规模化、配套化。进一步深化城市管理体制改革，推动管理工作重心下移，充分发挥街道、社区管理城市的积极性。继续深入开展“五化三创”工作，严格市容环境考评，建立市容市貌长效管理机制。经营好城市的土地和无形资产，筹集更多的资金用于城市建设；继续抓好项目融资，用好开行贷款，加强城镇基础设施建设；鼓励社会资本通过多种形式参与市政公用设施建设，有偿转让城市公益性基础设施的产权、经营权以及城市公用行业的特许经营权。积极抓好城市无形资产的市场化运作，稳步推进市政基础设施有偿使用。

　　切实加快小城镇建设。完善市域城镇体系规划。重点发展县城和规模较大的建制镇，引导乡镇企业和非农产业向小城镇聚集，走产业兴镇之路，增强城镇综合实力。加大小城镇建设投入，完成小城镇基础设施建设投资4.8亿元。认真贯彻落实户籍管理的有关政策。净增城镇人口12.7万人。继续实行重点镇动态管理，开展文明卫生城镇创建活动，提升城镇品位。

　　（四）进一步深化改革，大力发展开放型经济和民营经济

　　全力推进国有企业改革。把国有企业改革作为明年深化改革的重头戏来唱，作为攻坚战来打。理顺国有企业产权关系和职工劳动关系，着力于盘活存量、吸引增量、扩大总量、优化结构，加快国有企业改革步伐，坚决完成国企改革年度计划。强化目标责任，规范操作程序，通过出售、转让、并购、联合、股份制改造、民营嫁接等形式，实现国有企业产权主体多元化。采取多种形式剥离企业的社会职能。创新企业运行机制，完善企业法人治理结构。进一步深化国有资产管理体制改革，健全国有资产管理体制和监督体系。同时要抓好集体企业、乡镇企业的改革。

　　抓好以农村税费改革为重点的农村改革。取消除烟草以外的农业特产税，降低农业税率。把减轻农民负担与乡镇机构、农村教育体制配套改革结合起来，调整和完善县乡财政体制，减少财政供养人员；继续抓好乡镇消赤减债工作；精简村组干部，压缩村组开支；调整农村教育布局，精简教师队伍，从体制上稳定和减轻农民负担。稳定农村土地承包制度，完善土地流转办法，依法保护农民对土地承包经营的各项权利，切实保证农民的土地流转收益，促进农业适度规模经营。推进粮食流通体制市场化改革，实行对种粮农民的直接补贴。抓好农村全面小康示范村建设。

　　推进要素市场改革。运用丰富的资本市场产品，满足投资者多层次、多元化的投资需求。规范发展土地市场，全面推行国有土地收购储备出让制度，垄断土地一级市场，对经营性建设用地全部实行挂牌、招标、拍卖。提升技术、劳动力市场层次和水平。加快要素价格市场化，打破行业垄断和地区封锁，促进各类生产要素自由流动。

　　全面实施开放活市战略。把招商引资作为加快经济社会发展的重中之重，举全市之力、集全民之智来抓。建立健全招商引资目标责任制，努力营造招商引资人人有责的氛围。坚决兑现鼓励招商引资的奖励办法，充分调动广大干部群众招商引资的积极性。完善项目全程代理制，实行一站式服务，一个窗口审批。创新招商方式，增强招商效果。充分发挥专业招商队伍和招商引资网络的作用。坚持务实招商原则，切实提高签约项目的履约率。对深洽会签约的项目，跟踪落实，搞好服务，促使资金早日到位，项目尽快建成投产。组织好温州、宁波招商和境外招商几大专题招商活动。努力扩大对外经济贸易。加强出口基地建设，扶持优势出口企业发展，调整优化出口商品结构，扩大优势工业产品和绿色、特色农产品的出口规模。积极推动有条件的企业“走出去”，拓展发展空间。积极参与国际经济技术交流与合作，不断提高我市经济的外向度。

　　放手发展民营经济。全面清理限制民营经济发展的政策，完善配套措施，消除体制性障碍。放宽市场准入，凡是法律法规未禁入的行业和领域，民营经济都可以进入，凡是三资企业可以进入的行业和领域，民营经济都可以进入。完善公平竞争机制，在投融资、税收、土地使用和对外贸易等方面，对民营企业与公有制企业一视同仁。引导民营经济推进技术创新，提高管理水平，扩张规模，提高素质。努力营造良好的投资环境，进一步激发广大群众的创业热情，增加民间资本投入，加快民营经济发展。

　　（五）抓好财税工作，加大金融对经济的支持力度

加强财源建设。充分发挥财政在调控经济运行中的重要作用，运用政府引导性投资、贴息、财政补贴、专项扶持等手段，吸引民间资本、外来资本和信贷资本投入，培育各类市场主体；运用财税手段，推动所有制结构、产业结构、产品结构、技术结构、企业组织结构的战略性调整，形成新的财税增长点。进一步巩固基础财源，壮大支柱财源，开辟新兴财源，培育后续财源，努力做大财政“盘子”。

　　加强财税征管。加快“金税工程”建设。加强税法宣传，改进纳税服务，营造良好的税收征管环境。坚持依法征税，依法纳税，禁止擅自出台税收优惠政策，严禁越权减税、免税、缓税，维护税收法律法规、政策的统一性、权威性和严肃性。继续推行“以票管税”制度。要依法严征细管，严厉打击抗税、偷税、漏税、骗税行为，做到应收尽收。拓宽征管领域，加大对易流失税源及小税种的征管力度；突出工作重点，加强对重点地区、重点行业、重点税种、重点企业的征管。强化税务稽查，杜绝“关系税”、“人情税”，严惩以税谋私行为。

　　加强支出管理。继续抓好部门预算、“收支两条线”管理、国库集中支付、政府采购、工资统发等各项改革，健全公共财政体制。进一步深化财政支出管理制度改革，牢固树立量入为出、节俭办事的思想，坚决反对铺张浪费。科学、合理地配置有限的财政资源，确保国家机关的正常运转，确保工资的统一发放，确保社保资金的按时支付，把扶贫帮困资金列入预算。强化资金和项目管理，努力防范财政风险。

　　积极引导金融机构支持地方经济发展。支持金融机构适应国家信贷政策的调整，拓宽信贷渠道，改进金融服务，优化信贷结构，扩大有效投入。加强银企合作，找准我市产业发展与信贷支持的结合点，召开各种形式的银企洽谈会、贷款营销会，加大贷款投放力度。突出扶优扶强，优先保证重点建设项目和重点企业的信贷需求。发挥农村信用社支持“三农”的主渠道作用，加大对县域经济的信贷投入，积极扩大农户小额信用贷款。加强对中小企业的信贷支持。支持金融机构深化改革、转变机制，增强市场竞争力和抗风险能力。大力创建金融安全区，有效防范和化解金融风险。

　　（六）全面发展各项社会事业，促进三个文明协调发展

　　深入实施科教兴市战略。振兴益阳，关键在科技，基础在教育，根本在人才。加强科普宣传教育，增强全体市民的科技意识。高度重视科技成果的转化推广，促进科技与经济的紧密结合，提高科技进步对经济增长的贡献率。大力发展中小型科技企业和民营科技企业。建立科技发明奖励制度，提高科技创新能力。大力引进科技人才和科技项目。建设好益阳市生产力发展中心和湖南省科技专家交流中心。努力构建现代国民教育体系和终身教育体系，建设学习型社会，增强受教育者的就业、创新和创业能力。继续巩固和提高“两基”成果。加快发展高中阶段教育。积极发展高等教育，支持办好湖南城市学院、湖南工艺美术职业学院，努力办好益阳职业技术学院，积极申办医学专科学校。大力发展民办教育。全面推进素质教育。继续抓好教育结构调整，优化教育资源配置，努力提高教育质量。加大教育投入，确保教师工资发放和学校正常运转。加强学校危房改造。加强教师队伍建设，完成教师核编定岗分流工作。加强教育软环境建设，规范教育监管行为。认真整治学校周边环境。坚决治理中小学乱收费。创新人才工作机制，培养、吸引和用好各类人才。适应加快发展的需要，重点培养和引进一批高层次和高技能人才，选拔一批本科以上优秀毕业生到益阳工作。着力打造一支优秀企业家队伍。完善激励制度，实行公平竞争，形成优秀人才脱颖而出和人尽其才的良好环境。建立和完善人才市场体系，进一步促进人才流动。

　　全面发展各项社会事业。加强文化基础设施建设，扶持发展文化公益事业。繁荣文艺创作，多出精品力作。创新文化活动，丰富人民群众的文化生活。切实做好疾病预防控制工作，建立健全应对公共卫生突发事件的长效机制。全面推进医疗卫生体制改革，提高人民卫生健康水平。加强医疗卫生队伍行业作风建设，强化医疗安全管理。加强卫生监督执法工作。广泛开展全民健身活动，做大做强羽毛球等优势项目，提高竞技体育水平。承办好2004年中国拳击公开赛暨雅典奥运会亚洲资格选拔赛。认真组建益阳市体育代表团参加湖南省第二届大众运动会。切实做好老龄、档案、地方志等工作。进一步发展其他各项社会事业。

　　落实可持续发展的各项措施。继续抓好计划生育工作，严格目标管理，稳定低生育水平，提高出生人口素质。严格土地管理，提高土地利用效率。突出抓好基本农田保护，确保耕地占补平衡，提高粮食综合生产能力。加强矿产、林业、水资源保护和合理开发。狠抓城市环境综合整治和生态环境保护，加大污染防治力度，强化环境监督管理，提高环境质量。

　　加强民主法制建设和精神文明建设。积极推进民主政治建设。各级政府要自觉接受人大及其常委会的监督，认真执行人大及其常委会的决议、决定。自觉接受政协的民主监督，广泛听取民主党派、工商联、无党派人士的意见，搞好重大决策的政治协商。高度重视人大代表建议、批评、意见和政协委员提案办理工作。完善民主决策程序，增强决策科学性。积极扩大基层民主，切实加强社区建设，完善村（居）民自治和企业民主管理制度。认真落实党的民族宗教政策，做好侨务、对台等工作，积极支持工会、共青团、妇联等人民团体的工作。着力推进依法治市战略，继续深入开展“四五”普法教育，增强全民法律意识。认真贯彻《公民道德建设实施纲要》，强化社会公德、职业道德和家庭美德教育。以“创星”为龙头，广泛开展各种形式的群众性精神文明创建活动。加强文化市场管理，倡导文明健康的生活方式，提高公民素质。充分发挥广播、电视、报纸等新闻媒体的导向作用，为加快经济发展，全面建设小康社会提供舆论保障。

　　努力维护社会稳定。落实社会治安综合治理各项措施，依法严厉打击各类犯罪活动特别是多发性犯罪和地下六合彩赌博活动，切实提高破案率，打击“法轮功”等邪教组织。坚持“打防结合、以防为主”，不断深化巡防社区警务体制改革，开展创建平安社区活动，控制违法犯罪。抓好不稳定因素的排查，认真研究应对措施，妥善处理各类突发性事件。继续下大力气抓好农村“两会一部”的清理整顿工作。高度重视和加强人民群众来信来访工作，严格落实领导信访接待日制度和信访责任追究制度，努力把人民内部矛盾化解和处置在萌芽状态。切实抓好安全生产和消防工作，避免重特大安全事故的发生。

　　加强国防后备力量建设，做好民兵预备役、国防动员和人民防空工作。深入开展拥军优属、军民共建活动，巩固军政军民团结。做好征兵和转业、退伍军人安置工作。

　　（七）做好就业和社会保障工作，不断提高人民生活水平

　　抓好就业再就业工作。就业是民生之本。把扩大就业放在经济社会发展更加突出的位置，努力改善创业和就业环境。坚持劳动者自主择业、市场调节就业和政府促进就业的方针。大力发展劳动密集型产业，创造更多的就业岗位。注重抓好劳动力市场和乡镇、街道、社区劳动保障平台建设。积极探索和推行灵活多样的就业方式。完善再就业目标管理责任制，落实再就业政策，强化再就业服务，加大再就业资金投入，加强职业教育和技能培训，帮助特殊困难群体就业，提高再就业率。

完善社会保障体系。完善城镇职工基本养老保险制度，狠抓养老保险扩面和基金征缴，大力推进社会化管理服务，确保养老金按时足额发放。健全失业保险制度，实现国有企业下岗职工基本生活保障向失业保险并轨。全面推行城镇职工基本医疗保险制度，停止实行公费医疗和劳保医疗，扩大基本医疗保险覆盖面，健全社会医疗救助和多层次的医疗保险体系。全面启动职工工伤保险和生育保险。完善城市居民最低生活保障制度，加大财政投入力度，提高保障水平，严格“低保”申请、核准、审批、发放程序，努力做到应保尽保。

　　提高人民生活质量。千方百计增加城乡居民收入，进一步提高城乡居民吃穿用等基本消费水平。加强食品药品安全监督管理工作，保证食品药品安全。加快旧城改造步伐，改善住房供应，努力解决中低收入家庭住房困难；搞好农村住宅建设的规划和管理，改善农村住房条件。加强公共交通、自来水、公园、青少年活动场所、老年公寓、福利院等公共、公益设施建设，改善城乡居民的生活环境。加强妇幼保健工作，搞好血吸虫病等地方病和传染病、职业病的防治，完善农村初级卫生保健网络，提高医疗保健水平。

　　做好扶贫帮困工作。继续开展对口开发式扶贫，巩固扶贫成果。做好灾民安置和移民工作，切实解决灾民和移民的生产生活问题。依法按政策解决失地农民和城镇拆迁户的补偿安置问题，探索建立城乡社会救助制度，努力扶助弱势群体。切实做好残疾人工作。积极发展社会福利、社会救济、优抚安置、社会互助和慈善事业。

　　（八）切实转变政府职能，加强政府自身建设

　　坚持依法行政，建设法制政府。政府工作必须坚持有法必依，执法必严，违法必究。认真贯彻实施《行政许可法》，全面清理不符合行政许可法规定的行政许可，加快配套制度建设。依法规范政府行为，逐步实现政府机构编制、行政程序、行政执法责任、政府投资行为的法定化。推行行政执法责任制，建立评议考核制、错案追究制，及时纠正违法和不当的行政行为。适时推进相对集中行政处罚权，解决多重多头执法问题。加强对政府工作人员，特别是行政执法人员的教育、管理和监督，提高依法行政和文明执法水平。政府工作人员特别是领导干部要切实增强法制观念，努力提高依法办事能力，成为遵纪守法和依法行政的模范。继续坚持把优化经济发展环境作为“一号工程”来抓，牢固树立搞僵搞崩不是本事、搞好搞成才是成绩的观念，善待外地客商，支持本地经济能人创业，服务纳税人。继续推行黄牌警告制度、责任追究制度和公开曝光制度，坚决查处行政不作为、乱作为行为，逐步实现优化经济发展环境由领导重视型、政府推动型向主动服务型、制度保障型转变。

　　转变政府职能，建设高效政府。深化行政管理体制改革，加快政府由管理型向服务型转变，切实实现政企、政事分开。逐步把政府职能转变到经济调节、市场监管、社会管理和公共服务上来。改革事业单位管理体制，发展行业协会和中介组织，逐步把政府的专业性、技术性、事务性工作转给事业单位、行业协会和中介组织。强化政府的科学决策和公共服务职能，完善社情民意反映渠道和制度，健全重大决策的社会公示制和听证制，推进决策的科学化、民主化。继续推进行政审批制度改革，进一步减少和规范行政审批。继续推进政务公开，实行“一个窗口对外”、“一站式”办公、“一条龙”服务。全面推行服务承诺制、限期办结制和过错追究制。大力精简控制会议，严格规范办事程序，全面推行行政效能监察，切实降低行政成本，努力提高工作效率。

　　坚持反腐倡廉，建设清廉政府。加强对政府工作人员，特别是对各级领导干部的反腐倡廉教育，树立正确的权力观、地位观和利益观，做到情为民所系、权为民所用、利为民所谋，清廉自守，克己奉公。认真落实廉政建设责任制、领导干部任期经济责任审计和述职述廉制度。巩固财政三项改革和联审会签制度成果。建立和完善经营性土地使用权出让招标拍卖、建设工程项目公开招投标、政府采购、产权交易进入市场等制度，从源头上防止和治理腐败。针对企业和群众反映强烈的各种问题，抓好专项治理，坚决纠正部门和行业不正之风。旗帜鲜明地反对腐败，对任何腐败分子都要彻底查处，严惩不贷。

　　坚持负重奋进，建设实干政府。政府工作人员要始终保持蓬勃朝气、昂扬锐气和浩然正气，大力弘扬“诚实守信，负重奋进”的益阳精神，艰苦奋斗，执政为民。提高公务员素质，努力建设一支政治坚定、业务过硬、作风扎实的公务员队伍。牢固树立新的政绩观，改进抓经济工作的评价和考核办法，主要看当地经济发展速度和质量提高没有，财政增收没有，招商引资项目落实没有，城乡居民收入增加没有，城市化率提高没有，经济社会发展是否协调。要与时俱进，锐意创新，勇于探索，敢于攻坚，创造性地开展工作，在高起点、高难度上开创新局面，争创新业绩。抢抓机遇，力争主动，以只争朝夕的精神，抓好经济建设和各项工作。迎难而上，坚韧不拔，积极应对各种挑战，顶着压力走，迎着困难上，不断向更高的目标攀登。勤政为民、求真务实、脚踏实地、真抓实干，力戒形式主义、官僚主义；深入实际，倾听群众呼声，关心群众疾苦，着力解决人民群众关心的热点难点问题。顾全大局，团结奋斗，万众一心，加快发展。

　　各位代表！2004年，市政府将按照尽力而为、量力而行、有所作为的原则，精心组织，强化措施，落实责任，努力办好人民群众普遍关心和迫切需要办理的10件实事：一是创建省级卫生城市。二是城镇新增3万人就业，支持引导农村劳务输出转移80万人。三是完成50%的国有企业改革任务。四是加快“一区两园”建设，完成高新区南扩跨铁路线高架桥、梅林路、云山路、银城路、团圆路、迎宾路、长春路工程建设。五是重点支持建设8所农村中心卫生院，办好农村新型合作医疗试点。六是完成400公里县乡公路改造，完成益阳至沅江公路、南县至茅草街公路建设，开工建设沅江至茅草街公路、桃江至马迹塘公路、马路口至奎溪公路。七是建设好市一中新校区，在中心城区支持改造建设两所小学。八是办好市特殊学校，资助聋哑盲孩子入学。九是完成秀峰湖、会龙山公园二期工程建设。十是完成中心城区天然气管道工程建设。

　　各位代表，当前益阳正处在加快发展的关键时期，我们面临的任务十分繁重，肩负的使命无比光荣。让我们紧密团结在以胡锦涛同志为总书记的党中央周围，高举邓小平理论伟大旗帜，全面贯彻“三个代表”重要思想，在中共益阳市委的领导下，团结和依靠全市人民，解放思想，与时俱进，励精图治，奋发有为，努力夺取我市改革开放和社会主义现代化建设的新胜利。
